# Mapping monoclonal anti-SARS-CoV-2 antibody repertoires against diverse coronavirus antigens

**DOI:** 10.3389/fimmu.2022.977064

**Published:** 2022-09-02

**Authors:** Matheus Oliveira de Souza, Bharat Madan, I-Ting Teng, Aric Huang, Lihong Liu, Ahmed S. Fahad, Sheila N. Lopez Acevedo, Xiaoli Pan, Mallika Sastry, Matias Gutierrez-Gonzalez, Michael T. Yin, Tongqing Zhou, David D. Ho, Peter D. Kwong, Brandon J. DeKosky

**Affiliations:** ^1^ Department of Pharmaceutical Chemistry, The University of Kansas, Lawrence, KS, United States; ^2^ Ragon Institute of Massachusetts General Hospital (MGH), Massachusetts Institute of Technology (MIT), and Harvard, Cambridge, MA, United States; ^3^ Vaccine Research Center, National Institute of Allergy and Infectious Diseases, Bethesda, MD, United States; ^4^ Aaron Diamond acquired immunodeficiency syndrome (AIDS) Research Center, Columbia University Irving Medical Center, New York, NY, United States; ^5^ Department of Medicine , Division of Infectious Diseases, Columbia University Irving Medical Center, New York, NY, United States; ^6^ Department of Biochemistry and Molecular Biophysics, Columbia University, New York, NY, United States; ^7^ Department of Chemical Engineering, The University of Kansas, Lawrence, KS, United States; ^8^ Department of Chemical Engineering, Massachusetts Institute of Technology, Cambridge, MA, United States

**Keywords:** SARS-CoV-2, yeast display, betacoronaviruses, SARS-CoV-2 variants, cross-reactive antibodies

## Abstract

Variants of severe acute respiratory syndrome coronavirus 2 (SARS-CoV-2) have emerged continuously, challenging the effectiveness of vaccines, diagnostics, and treatments. Moreover, the possibility of the appearance of a new betacoronavirus with high transmissibility and high fatality is reason for concern. In this study, we used a natively paired yeast display technology, combined with next-generation sequencing (NGS) and massive bioinformatic analysis to perform a comprehensive study of subdomain specificity of natural human antibodies from two convalescent donors. Using this screening technology, we mapped the cross-reactive responses of antibodies generated by the two donors against SARS-CoV-2 variants and other betacoronaviruses. We tested the neutralization potency of a set of the cross-reactive antibodies generated in this study and observed that most of the antibodies produced by these patients were non-neutralizing. We performed a comparison of the specific and non-specific antibodies by somatic hypermutation in a repertoire-scale for the two individuals and observed that the degree of somatic hypermutation was unique for each patient. The data from this study provide functional insights into cross-reactive antibodies that can assist in the development of strategies against emerging SARS-CoV-2 variants and divergent betacoronaviruses.

## Introduction

The severe acute respiratory syndrome coronavirus 2 (SARS-CoV-2) is an enveloped, positive-sense single-stranded RNA virus that belongs to the sarbecovirus subgenus of the betacoronavirus (β-coronavirus) genus (1–3). SARS-CoV-2 is the etiological agent of Coronavirus Disease 2019 (COVID-19) that has caused over 500 million cases and 6 million deaths to date throughout the ongoing pandemic (4, 5). SARS-CoV-2 presents high transmissibility (6) with an estimated reproductive number R_0_ of 3.1, which is more contagious than several other respiratory viruses including SARS-CoV (R_0_ = 0.58), MERS (R_0_ = 0.69) and Influenza (R_0_ = 1.27) (7). In addition to SARS-CoV-2, six other coronaviruses are known to infect humans. Four of these (HCoV-229E, HCoV-OC43, HCoV-NL63, and HCoV-HKU1) circulate annually and generally cause mild upper-respiratory symptoms in individuals (8–10). The Severe Acute Respiratory Syndrome Coronavirus (SARS-CoV), and Middle East Respiratory Syndrome Coronavirus (MERS-CoV) are human coronaviruses that have resulted in two epidemics, SARS in 2002-2003 with a fatality rate of about 10%, and MERS in 2012, which presents a high case-fatality rate of 36% (11–13). The possibility of the emergence of a novel coronavirus with a high transmissibility as of SARS-CoV-2 and high fatality rate as of MERS brings high urgency for tools to track the impact of SARS-CoV-2 mutations and variants on immune responses, and clinical interventions to inform the development of new pan-betacoronavirus countermeasures (14).

The recent emergence of SARS-CoV-2 into human populations, combined with its rapid spread and comparatively high reproduction rate, have combined to fuel continual diversification of genetic variants since the earliest phases of the pandemic in 2019. The emergence of genetic changes has resulted in drastic phenotypic differences in transmission rates, virulence, and viral susceptibility to targeted biologic interventions including monoclonal antibody therapies and vaccines (15, 16). The World Health Organization (WHO) classifies SARS-CoV-2 variants in three different groups based on phenotypic characteristics. Variants of concern (VOCs) have characteristics of increased transmissibility, increased disease severity, and a measurable impact on countermeasures as diagnostics and vaccines (17–19). As of June of 2022, Omicron is the only currently circulating VOC. The Omicron variant (B.1.1.529), first detected in South Africa in November 2021, showed a high number of mutations and an extremely rapid global spread. Omicron variant showed much higher transmissibility than the original strain of SARS-CoV-2, concurrently with decreased vaccine efficacy and reduced susceptibility to monoclonal antibodies and passive serum antibody transfer from convalescent patients (20, 21). A similar trend was previously observed with the rise of the Delta variant (B.1.617.2), which was first identified in India in October 2020, and rapidly became the dominant variant in many regions around the globe (22, 23). Other VOCs that circulated previously include the Alpha variant (B.1.1.7), first detected in the United Kingdom in September 2020; the Beta variant (B.1.351), first documented in South Africa in May 2020; and the Gamma variant (P.1), first observed in Brazil in November 2020 (24–26). Another classification for the SARS-CoV-2 variants is as Variants of Interests (VOIs). VOIs present changes to their structure that can affect virus characteristics and present an emerging risk to public health due to high transmission (17, 19). Examples of VOIs include the Epsilon variant (B.1.427/B.1.429) that was detected in USA in March of 2020 (27). The third category that variants can be classified according to WHO is as Variants under Monitoring (VUMs) (17, 19). These variants present mutations that have the potential of affecting virus characteristics, but unclear evidence of how they affect transmissibility, virulence, and effectiveness of diagnostics, vaccines, and therapeutics. As SARS-CoV-2 continues to evolve, new variants will continually emerge. The structural and functional changes can greatly impact adaptive immune recognition, and a major goal for our scientific community is to understand, and potentially predict, how the emergence of new genetic variants can impact established immune memory elicited by exposure to previous SARS-CoV-2 strains.

The development of clinical interventions has struggled to keep pace with the rapidly diversifying strains of SARS-CoV-2. Numerous vaccine candidates have been developed throughout different phases of clinical trials, encompassing a broad variety of vaccine technology platforms including nucleic acids, inactivated virus, viral vectors, live attenuated virus, virus-like particles, recombinant proteins, and peptides (28–31); the vast majority of these vaccines, and all vaccines clinically approved by the US FDA, are designed from the original emergent SARS-CoV-2 strain first published in January 2020. For the treatment of the disease, one small molecule antiviral drug (remdesivir) has been approved by the FDA, and Emergency Use Authorizations (EUAs) have been issued to two other drug treatments (nirmatrelvir+ritonavir, and molnupiravir) and to two monoclonal antibodies (mAbs) – Evusheld and Bebtelovimab (32–36). Before the COVID-19 pandemic, only two monoclonal antibodies had received approval from the FDA for the treatment of infectious diseases: palivizumab targeting respiratory syncytial virus (RSV), and ibalizumab to treat HIV infection (37–39). Many therapeutic monoclonal antibodies against COVID-19 neutralize SARS-CoV-2 infection by binding to the viral Spike glycoprotein and preventing the interaction between the SARS-CoV-2 and its entry receptor, angiotensin-converting enzyme 2 (ACE2) (40, 41). The Spike glycoprotein comprises the subunits S1, responsible for ACE2 receptor binding, and S2, which mediates membrane fusion (42, 43). Antibodies that protect by disrupting viral attachment to ACE2 have also been identified against other betacoronaviruses, and distal regions from the S1 receptor-binding subdomain are promising targets for broadly neutralizing pan-betacoronavirus antibodies, especially against the conserved stem helix within the S2 subdomain (44–46). While most clinical antibody development efforts have focused on neutralizing antibodies (nAbs), it has been shown that cross-reactive antibodies are often non-neutralizing but can offer protection through important effector mechanisms, which include complement-dependent cytotoxicity, opsonization, antibody-dependent cellular cytotoxicity (ADCC) and antibody-dependent cellular phagocytosis (ADCP) (47–50). Thus, the characterization of antibody cross-reactivity against broad SARS-CoV-2 genetic variants provides a critical window on the molecular basis of recognition and immune memory against current and future circulating strains.

Despite the importance of understanding established antibody immune memory against emerging variants, it remains technically complex and resource-intensive to analyze naturally elicited antibody binding and cross-reactivity against broad SARS-CoV-2 antigen panels (51–54). Major issues include the need for viable primary cells for every antigen sort, and the necessity to express each antibody as a monoclonal for soluble plate-based assays. Recent technologies provide a remarkable advance and even permit the high-throughput screening of panels of antigens against primary cells (55, 56); however, the primary cells are lost after the initial analysis and so library of antibodies cannot be screened against future emergent variants. To directly address these questions related to the analysis of established antibody immune memory against future evolving viral strains, we applied a recently developed technology to immortalize native antibody immune repertoires as yeast display libraries (57–60) for renewable screening against both current and future emergent strains. In this study, we combined renewable yeast display library screening with coronavirus Spike antigen probe panels (61, 62) to follow the responses of convalescent COVID-19 patients against continually evolving SARS-CoV-2 variants and other broadly recognized betacoronavirus antigens. We show that natively paired antibody yeast display screening can reliably detect antibody subunit binding specificity and cross-reactivity using NGS data mining of sorted yeast display library populations, enabling efficient determination of antibody-antigen recognition for large antigen panels on a repertoire scale. Moreover, we found that the vast majority of cross-reactive mAbs elicited are non-neutralizing and present a repertoire-scale comparison of SARS-CoV-2-specific antibodies that show less mutations than donor-matched B cell repertoires on average, even after primary exposure with severe disease. These data reveal previously unreported genetic and phenotypic features of established human antibody immune memory against emergent future SARS-CoV-2 variants on a repertoire scale.

## Materials and methods

### Human sample collection

Peripheral blood mononuclear cells (PBMCs) were collected from two COVID-19 convalescent patients at Columbia University Irving Medical Center in New York, NY under IRB #AAAS9722, with informed consent obtained prior to sample collection. Donor 1 was a 54 year-old patient whose blood sample was collected in April 2020, two weeks after hospital admission. Donor 2 was a 71 year-old patient whose blood sample was collected in June 2020, three weeks after hospital admission.

### Emulsion-based overlap extension RT-PCR and yeast display antibody library generation

B cells were isolated from cryopreserved PBMCs, and natively paired heavy and kappa light chain variable region amplicons were amplified and cloned into a yeast surface display vector as described previously (57, 63–65). Briefly, memory B cells were isolated from patient PBMCs by magnetic bead separation and stimulated *in vitro* for 5 days for stimulation using IL-2, IL-21, and co-culture with fibroblast cells expressing CD40L to aid in B cell activation and expansion (63, 66). Using a flow focusing device, single B cells were captured in emulsion droplets, lysed, and oligo (dT)-coated magnetic beads were used to capture mRNA from single B cells (63, 64, 67). Beads were washed and used as template for an overlap extension reverse transcription PCR (OE RT-PCR) to link heavy and light chains onto the same cDNA strands. Two restrictions sites (NheI and NcoI) were incorporated into the OE linker region between heavy and light chains to enable downstream cloning into display libraries (57). To determine the diversity of the resulting cDNA samples, next-generation sequencing (NGS) was performed by 2×300bp Illumina MiSeq (64). cDNA amplicons encoding heavy and kappa light chain variable regions were amplified with primers containing the restriction enzymes AscI and NotI, and cloned into a yeast display vector for human Fab surface expression (57). The libraries were digested, ligated, transformed into high efficiency electrocompetent *E. coli*, and maxi-prepped. Next, plasmid libraries were digested with NheI and NcoI, and ligated with an insert containing a pre-digested bidirectional Gal1/Gal10 promoter. The resulting ligated product was transformed into electrocompetent *E. coli*, and maxi-prepped and PCR amplified prior to the transformation by homologous recombination into yeast strain AWY101 (57). The yeast transformed libraries were passaged twice in SD-CAA (20 g/l dextrose, 6.7 g/l yeast nitrogen base, 5 g/l casamino acids, 8.56 g/l NaH_2_PO_4_.H_2_O, and 10.2 g/l Na_2_HPO_4_.7H_2_O) media to ensure single plasmid in each yeast cell.

### SARS-CoV-2 and broad betacoronavirus probe production and isolation

Betacoronavirus antigen probes were produced as described previously (61, 62). Briefly, DNA sequences of SARS-CoV-2 and broad coronavirus spike glycoprotein, RBD or NTD subdomains were cloned into an expression vector and an AVI tag was added after the C terminus for biotinylation. Transient transfection of 293 Freestyle cells, affinity purification, tag-cleavage, and biotinylatiion were carried out, followed by column purification and characterization of the probes by SDS-PAGE.

### Yeast antibody library screening against betacoronavirus antigens *via* FACS

Yeast antibody libraries were incubated for 36 hrs in SGDCAA (SD-CAA media with 2g/l dextrose and 20 g/l galactose) at 20°C, 225 rpm to induce Fab surface expression. FACS sort gates were drawn for sorting as previously described (57, 58, 60, 68) and evaluated using a Sony MA-900 flow cytometer with sort capability. For the first round of enrichment, 3 × 10^7^ yeast cells were washed twice with ice-cold staining buffer (1× PBS with 0.5% BSA and 2 mM EDTA) and incubated with anti-FLAG FITC Clone M2 for 30 minutes. Yeast cells that expressed Fab on the surface, referred as VL+, were collected, expanded in SD-CAA (pH 4.5) for 24–48 h at 30°C and transferred to SGD-CAA media at 20°C for a second round of screening. Fab expression was detected using the anti-VL FITC marker, and antigen binding was detected with either allophycocyanin (APC) or phycoerythrin (PE) channels conjugated to betacoronavirus antigen probes, as previously described (61, 62).

For Fab recognition screening against SARS-CoV-2 variant probes, VL+ enriched libraries were enriched for three rounds against 20 nM SARS-CoV-2 S2P (stabilized spike-derived ectodomain) probes of SARS-CoV-2 Alpha (B.1.1.7), Beta (B.1.351), Gamma (P.1), Delta (B.1.617.2) and Epsilon (B.1.427) variants. Biotinylated antigen probes were pre-conjugated to streptavidin-APC (SA-APC, Thermo Fisher Scientific) in a 4:1 molar ratio (antigen:fluorophore) as previously described (69). The monoclonal anti-FLAG-FITC antibody was also included to measure Fab expression of the yeast libraries concurrently. The mixture containing the antigen probe, anti-FLAG-FITC, and yeast cells were incubated for 30 minutes, washed 3 times with staining buffer (1× PBS with 0.5% BSA and 2 mM EDTA), and sorted *via* FACS.

To test Fab recognition of SARS-CoV-2 variant subdomains, VL+ enriched libraries were stained with 20 nM of WA-1 SARS-CoV-2 S2P probe for two rounds of enrichment, and then screened for binding against 100 nM receptor binding domain (RBD) and 100 nM N-terminal domain (NTD) antigen probes from the SARS-CoV-2 Alpha, Beta, Gamma, Delta and Epsilon variants. Biotinylated antigen probes were pre-conjugated to SA-APC in a 4:1 molar ratio (antigen:fluorophore). Monoclonal anti-FLAG-FITC and yeast cells were added to the mix, which was incubated for 30 minutes, washed 3 times with staining buffer, and sorted *via* FACS.

For Fab recognition screening against broadly recognized betacoronavirus probes, VL+ enriched libraries were stained with 20 nM of D614G S2P probe for two rounds of enrichment, and then screened for binding against 20 nM broad betacoronavirus trimer probes of HCoV-OC43, HCoV-HKU1, SARS-CoV, MERS, WIV-1 and SCH014 for one round. Staining was performed as described above, using Streptavidin conjugated to R-Phycoerythrin (SA-PE, Thermo Fisher Scientific, Waltham, MA, USA).

The Sony MA-900 cell sorter was used for screening of the yeast libraries, along with associated software for sort data analysis. After each sort round, samples were expanded 24–48 h at 30°C and sequenced as described below. For all sorted samples, the VL+ samples were used as reference for enrichment ratio (ER) calculations. Flow cytometry data were analysed using FlowJo 10.4 (FlowJo, LLC, Oregon, USA).

### NGS data collection and bioinformatic analysis of sorted yeast libraries

Sorted yeast samples were cultured after each round of FACS as described above, and yeast plasmid DNA was extracted using a high efficiency protocol described previously (57, 60, 70). Heavy chain genes from each library were amplified using primers targeting the yeast display vector backbone using a high-fidelity polymerase (Kapa Hifi HotStart Mastermix, Kapa Biosystems, MA). Next, a second round of PCR was performed to add a unique barcode identifier to each amplicon library for sequencing on an Illumina 2x300 MiSeq platform. Illumina sequences were quality-filtered as previously described (60). The frequency (F) for a given CDR-H3 sequence (x) in a sorted sample (y) and the enrichment ratio (ER) of each CDR-H3 compared to the Fab-expressing (VL+) antibody library (ER_x,y_) for each clone were calculated to track the functional performance of each clone *x* across antigen sort group *y* (60). ER represents the ratio of the frequency of each clonal lineage CDR-H3 compared to the Fab-expressing (VL+) antibody library.

$F_{x,y}=\frac{reads_{x,y}}{sum\;of\;reads\;in\;y}$ $ER_{x,y}=\frac{F_{x,y}}{\;F_{x}\;in\;VL^{+}sample\;}$

Comprehensive functional analysis of sorted antibody libraries was performed *via* bioinformatic interrogation of NGS data. We determined clones that are specific to SARS-CoV-2 by analyzing the variant ER, with a minimum of 10 NGS reads in the second round of enrichment against SARS-CoV-2 WA-1 S2P (57, 60). We assessed RBD and NTD subdomain sorts data to predict epitopes recognized by selected antibodies, based on the antigen with the highest ER for a given monoclonal antibody target. Statistical significance for somatic hypermutation (SHM) data was evaluated using the Kolmogorov-Smirnov test in Prism 7 Software, GraphPad SHM plots were generated using the same software (60).

### mAb expression and purification

Antibody heavy and light chain variable region genes were inserted into IgG1 mammalian expression vectors, as described previously (57). Heavy and light chain expression plasmids were co-transfected into Expi293F cells using an ExpiFectamine 293 Transfection Kit (Thermo-Fisher Scientific, MA) according to the manufacturer’s instructions. After harvest at day 6 of transfection, IgGs were purified using a Protein G resin (GenScript, NJ), concentrated with an Amicon Ultra-4 Centrifugal 30 kDa filtration unit (MilliporeSigma, MD), and stored at 4°C.

### ELISA against betacoronavirus antigens

96-well EIA/RIA ELISAplates (Corning, NY) were coated with 100 ng/well SARS-CoV-2 variants probes when testing for affinity, or 100 ng/well lysozyme from chicken egg white (MilliporeSigma, MD) when testing for polyreactivity. Coating was performed at 4°C overnight, followed by blocking with 200 µL of blocking buffer (5% w/v BSA, 0.1% v/v Tween-20 in PBS) for 2 hours at room temperature. Purified antibodies were added to plates and incubated for 2 hours at room temperature after being serially diluted in blocking buffer. Plates were incubated with a 1:2,000 dilution of Mouse anti-human IgG Fc HRP conjugate secondary antibody (A18853, Invitrogen, Carlsbad, CA) in blocking buffer at room temperature for 1 hr. PBST (0.5% Tween-20 in PBS) was used to wash plates between each step. Next, 100 ml of Super AquaBlue ELISA substrate (Thermo Fisher) was added and incubated for 10 min before the reaction was stopped using 0.625 M oxalic acid. A Synergy H1 plate reader (BioTek) was used to measure absorbance at 405 nm. All ELISA experiments were performed in duplicate.

### Pseudovirus neutralization assays

Constructs of the Spike protein of SARS-CoV-2 D614G and SARS-CoV were produced and confirmed by sequencing to generate pseudovirus particles, as previously described (21, 71–73). Lipofectamine 3000 (ThermoFisher, MA) was used to transfect the constructs into Human Embryonic Kidney (HEK) 293T cells. Cells were washed after 24 hrs of transfection with complete medium (DMEM + 10% FBS + penicillin/streptomycin), infected for 2 hours with rVSV-G-pseudotyped ΔG-luciferase (G*ΔG-luciferase, Kerafast), and incubated at 37 °C under 5% CO2 for 24 hrs. The supernatant with pseudoviruses was collected and clarified by centrifugation at 300 × *g* for 10 mins. The rVSV expressed SARS-CoV-2 pseudoviruses were then incubated with serial dilutions of purified antibodies. Reduction in luciferase gene activity was quantified and measured with a SpectraMax i3x Multi-Mode Microplate Reader using SoftMax Pro 7.0.2 for determination of neutralization.

## Results

### Generation of natively paired heavy and light chain yeast surface display libraries

**Figure 1:**
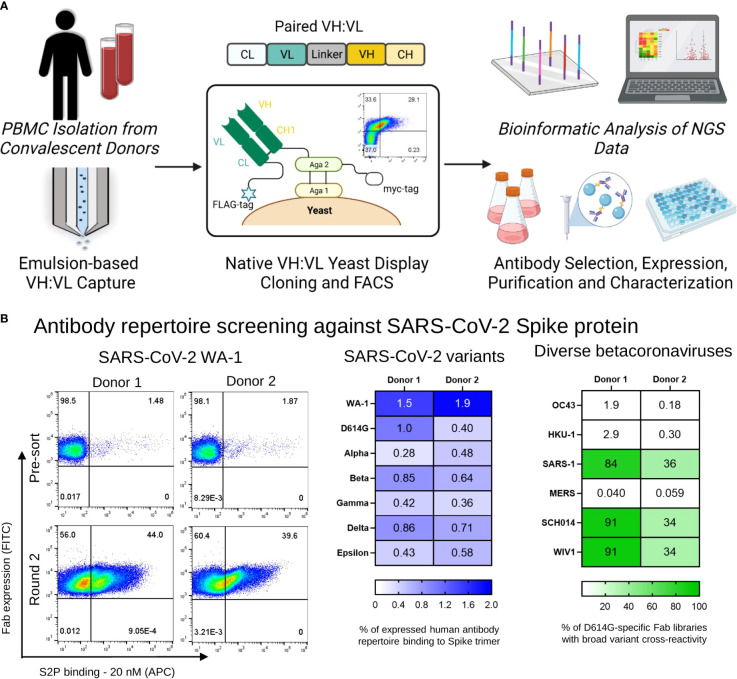
Functional screening of natively paired antibody repertoires from SARS-CoV-2 convalescent donors. **(A)** Natively paired yeast display library generation, FACS screenings and NGS-based antibodies selection and characterization. Peripheral memory B cells from two COVID-19 convalescent donors were isolated, and natively VH : VL antibody gene sequences were captured from CD27+ antigen-experienced B cells *via* emulsion overlap extension RT-PCR. Antibody libraries were constructed to clone the VH : VL antibody repertoire for yeast surface expression in a fragment antigen binding (Fab) format. Broadly recognized coronavirus probes were used for multiple rounds of fluorescence-activated cell sorting (FACS) screening to map antibody responses against SARS-CoV-2 Spike antigens. Sorted yeast was sequenced with Illumina Next-Generation Sequencing (NGS), and NGS data was mined to interpret functional characteristics for each anti-SARS-CoV-2 Spike antibody in the dataset. A subset of antibodies was selected for expression as full IgGs and *in vitro* characterization**. (B)** (*Left*) Representative FACS analysis of yeast display repertoires screened for binding to the SARS-CoV-2 WA-1 Spike protein. (*Center*) Heat map of the percentage of human antibody repertoire binding to SARS-CoV-2 Spike trimers that recognize WA-1, D614G, Alpha, Beta, Gamma, Delta, and Epsilon variants. (*Right*) Yeast display libraries were enriched for 2 rounds against the D614G Spike trimer antigen, and the percentage of anti-D614G mAbs that were cross-reactive to coronavirus Spike trimers of WIV1, SCH014, OC43, MERS, SARS-1, and HKU-1 by antigen staining are shown in green.

Cryopreserved peripheral blood mononuclear cells (PBMCs) were obtained from two COVID-19 convalescent donors for functional profiling of natively paired heavy and light variable regions (VH : VL) antibody repertoire. CD27+ selection was performed for enrichment of antigen-experienced B cells and the memory B cells were stimulated *in vitro* as previously described (66). A flow-focusing device was used to capture single B cells, followed by overlap extension RT-PCR to link VH : VL amplicons (57–60). From the stimulated cell population, 28,895 and 22,301 unique antibody lineage clusters were identified from Donors 1 and 2, respectively, using previously reported bioinformatic parameters (60, 63, 66). Paired heavy and light chain cDNA libraries were cloned into a yeast display vector that allowed expression of antibody Fabs on the surface of yeast for functional FACS screening and NGS analysis of sorted antigens (**Figure 1A**).

### FACS screening of yeast surface display libraries

We applied yeast surface display to screen the immune repertoire of two COVID-19 convalescent patients against broad SARS-CoV-2 variants and betacoronavirus probes (57, 60). Antibody yeast libraries were sorted to assess monoclonal antibody recognition breadth across SARS-CoV-2 variants and human and bat betacoronaviruses. For SARS-CoV-2, we evaluated binding against SARS-CoV-2 variants, using probe formats including the full S2P Spike protein, as well as the RBD and NTD subdomains (61, 62). For sorts involving the SARS-CoV-2 spike protein, three rounds of enrichment were performed, followed by sequencing of the sorted libraries. **Figure 1B** (S1) shows the percentage of binding of VL+ selected pre-sorted libraries against the Spike Trimer of each SARS-CoV-2 variant. Similar to previous reports (74), the majority of yeast antibody libraries showed reduced recognition of S2P variants compared to the earliest emergent SARS-CoV-2 strains that likely matched what the donors were exposed to in March and May of 2020 for Donors 1 and 2, respectively during their admission at Columbia University Irving Medical Center (**Figure 1B** *center*, **Figure S1**) For screening against SARS-CoV-2 subdomains, yeast display libraries were enriched for two rounds with 20 nM against the Spike protein of SARS-CoV-2 WA-1, followed by sorts with 100 nM RBD and 100 nM NTD for each variant in this study (**Figure S2**). We used spike protein probes for the human coronaviruses HCoV-OC43, HCoV-HKU1, SARS-CoV, and MERS for pan-betacoronavirus screening, including the bat coronaviruses WIV-1 and SCH014. Yeast libraries were enriched for two rounds with 20 nM SARS-CoV-2 D614G S2P, following staining with 20 nM of each broad betacoronavirus spike probe to determine cross-reactivities between D614G and other broad betacoronavirus antigens. We observed that both patient libraries presented D614G-specific antibodies that are cross-reactive to broad coronaviruses, specially to SARS-CoV, WIV-1 and SCH014, and the percentage of cross-reactive antibodies varied between 84-92% for Donor 1 and 34-36% for Donor 2 for those antigens (**Figure 1B** *right*). Next, we analyzed NGS data of the yeast libraries for all the sorts mentioned above to match antigen recognition features with monoclonal antibody lineages from established immune memory of convalescent COVID-19 patient memory B cells.

### Monoclonal antibody selection and binding analysis

**Figure 2:**
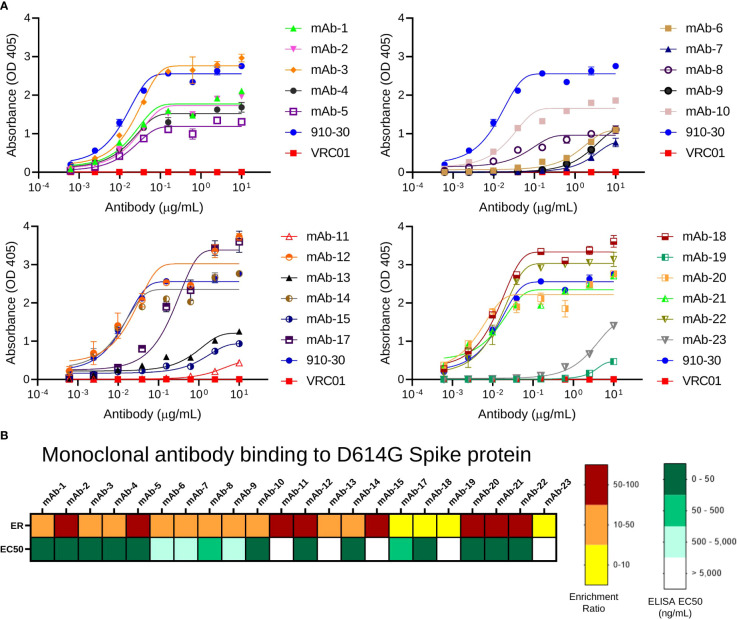
Validation of predicted antibody clones by ELISA. **(A)** ELISA absorbance profiles of expressed IgGs to SARS-CoV-2 D614G S2P protein. Data are represented as mean ± SEM. **(B)** Comparison between ELISA EC_50_ value and ER for anti-SARS-CoV-2 mAbs against SARS-CoV-2 D614G S2P.

Antibody sequences in pre-sort libraries and in sorted samples were determined using 2x300bp Ilumina MiSeq analysis. Antibody recognition was determined by tracking the CDR-H3 for each clone in sorted libraries, and a panel of 23 clones that presented higher ERs across SARS-CoV-2 variants and broad coronaviruses antigen sort experiment was selected for expression and evaluation as monoclonal antibodies. The VH and VL regions of the 23 selected antibodies were synthesized and cloned into human IgG1 expression plasmids. Antibodies were expressed in Expi293 cells as full-length IgGs and purified using protein G. Antibody binding was evaluated by ELISA against SARS-CoV-2 D614G Spike protein (**Figure 2A**), which revealed all antibodies expressed presented binding against their antigen probe as predicted by NGS and confirmed that the process of FACS, NGS data analysis, and bioinformatic interpretation of antigen-specific antibodies correlated well with biological binding assays for monoclonal antibody validation. One of the monoclonal antibodies (mAb-16) expressed with very low yield, and ELISA was not performed for this mAb. To further investigate the correlation between library screening data and ELISA results, we evaluated the correlation between ER and ELISA EC_50_ (half maximal effective concentration) for 23 antibodies evaluated (**Figures 2B** and **S3**). With this binding analysis, we did not observe statistically significant trends between ER and EC_50_ (r^2^ = 0.055, p = 0.29), which was similar to previous studies that showed that an ER>5 in Round 2 of antigen-binding sort gates was highly correlated with antigen recognition as monoclonal antibodies, but the magnitude of ER was not correlated with affinity in the absence of carefully defined affinity sort gate strategies (57, 60, 68).

### NGS-based analyses of anti-SARS-CoV-2 monoclonal antibody repertoires

**Figure 3:**
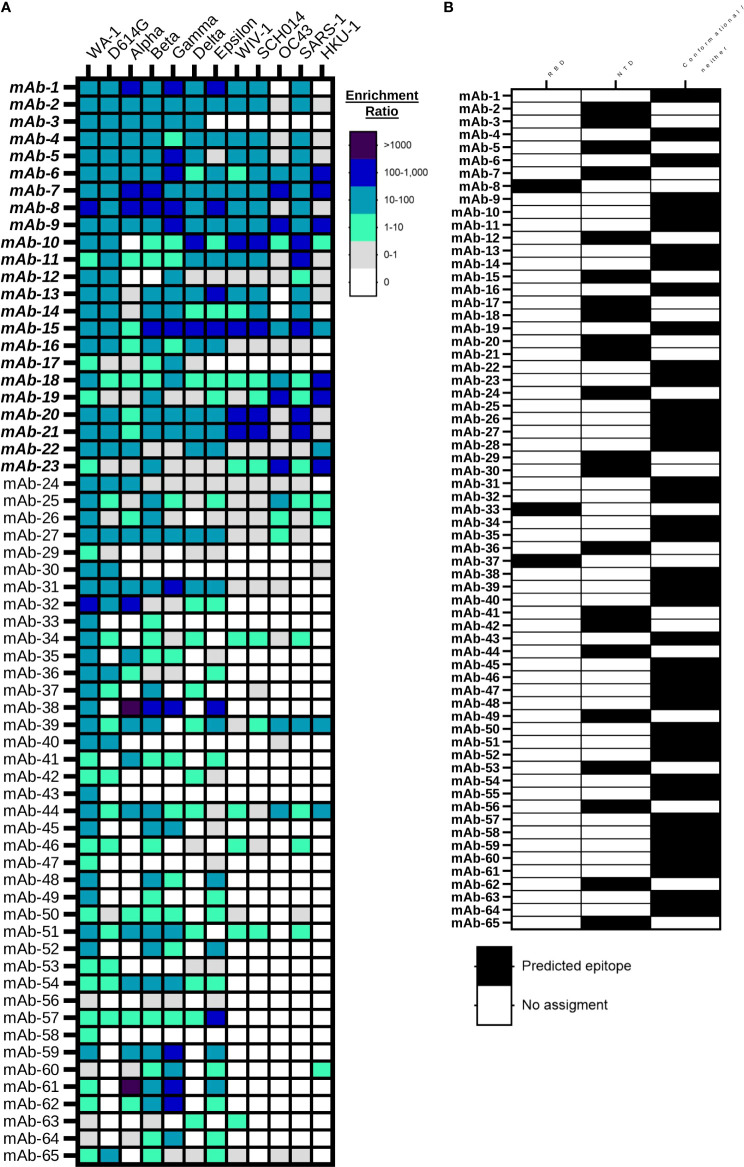
Large-scale betacoronavirus recognition breadth and epitope mapping analyses for anti-SARS-CoV-2 mAbs. **(A)** Bioinformatic analysis of yeast display library enrichment ratios determined by NGS after 2 rounds of enrichment against broad coronavirus S2P probes. Antibodies were screened against eight SARS-CoV-2 variants (SARS-CoV-2 WA-1, D614G, Alpha, Beta, Gamma, Delta, and Epsilon) and six broader betacoronaviruses (WIV-1, SCH014, OC43, SARS-1, MERS and HKU-1). Enrichment against MERS Spike protein was not observed, and is omitted here. **(B)** Bioinformatic analysis of monoclonal antibody recognition of SARS-CoV-2 variants RDB and NTD subdomain probes based on enrichment ratios in NGS sort data.

We performed large-scale mapping of monoclonal antibody responses against diverse SARS-CoV-2 and betacoronavirus antigens using NGS. First, we evaluated antibody specificity and cross-reactivity by analyzing the ER each antibody across different betacoronavirus probe sort conditions. We identified a total of 65 antibody linages that were specific to SARS-CoV-2 as defined by analysis of the variant ER, with a minimum of 10 NGS reads in the second round of clonal enrichment against SARS-CoV-2 WA-1 S2P antigen. Further, we applied an ER analysis to further identify cross-reactive antibody lineages against betacoronavirus antigens (**Figure 3A**). Cross-reactivity data show that decreased binding was observed against newly emerged SARS-CoV-2 variants relative to the Spike protein of the WA-1 early SARS-CoV-2 variant, but still, even samples collected in April and June of 2020 revealed the presence of numerous monoclonal antibodies with cross-reactivity against SARS-CoV-2 VOC, VOI, and diverse betacoronaviruses, many of which had yet to emerge at the time of sample collection in this study.

We also applied a high-throughput ER analysis to track the subdomain targets of antibody recognition through analysis of enrichment data against RBD and NTD for all the variants. We predicted the antibodies that are RBD-specific, NTD-specific, and the ones that do not bind to either soluble RBD or NTD probes, and therefore likely bound to conformational epitopes or other targets on Spike like the S2 domain (**Figure 3B**). We found that 3/65 (5%) of the cross-reactive antibodies in our screening data bound to the soluble RBD antigen, 22/65 (34%) of antibodies were specific to the soluble NTD antigen, and the rest of the antibodies (40/65 or 61%) bind to conformational epitopes or regions of the spike protein outside of RBD and NTD. To exclude the possibility of polyreactivity leading to antibody recognition against many different sorted antigens, we evaluated two of the antibodies that enriched against both RBD and NTD for polyreactivity using lysozyme from chicken egg white (**Figure S4**), which is a potential concern for antibodies encoded by human B cells (75). The antibodies tested (mAb-9 and mAb-22) did not show binding against lysozyme, suggesting that they likely bind to a conformational epitope with some recognition of both the RBD and NTD subdomains.

### Somatic hypermutation of anti-SARS-CoV-2 mAb repertoires

**Figure 4:**
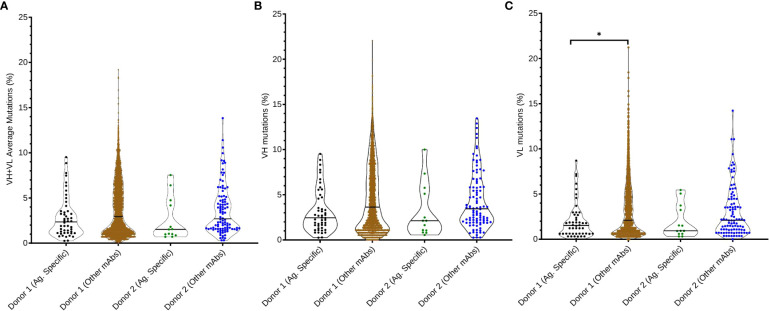
Somatic hypermutation analysis of anti-SARS-CoV-2 Spike antibodies compared with non-SARS-CoV-2 Spike antibodies in donor immune repertoires. **(A)** Average SHM percentage of both VH and VL genes in the SARS-CoV-2 Spike-specific repertoire, compared with the rest of the donor’s immune repertoire. Percentage of somatic hypermutations in **(B)** heavy chain variable regions and **(C)** light chain variable regions is also reported for each donor and dataset. Pairwise comparisons were performed using the K-S test, and all statistically significant comparisons are noted (*p< 0.0083 after correcting for multiple comparisons using Bonferroni correction); any non-statistically significant comparisons are omitted.

We evaluated the extent of somatic hypermutation (SHM) for SARS-CoV-2 WA-1 strain antigen-specific sequences compared to non-antigen specific sequences from the two convalescent human donors (**Figures 4A–C**). We observed no statistically significant differences between SHM values according to antigen specificity, except for SARS-CoV-2 antigen-specific VL mutations compared to the rest of the antibody immune repertoire of Donor 1 (p = 0.0054). However, SARS-CoV-2-specific antibodies consistently showed lower average levels of SHM than other antibodies in the patient repertoire, for all comparisons evaluated (VH : VL genes, VH genes separately, and VL genes separately). These data suggested that B cells at the time of sampling had gone through fewer rounds of B cell clonal expansion and selection than for the average B memory response at the time of sample collection in these two COVID-19 convalescent donors.

### Neutralization analyses for anti-SARS-CoV-2 antibodies

We assessed the neutralization potency for the panel of 23 antibodies that were expressed as IgG in this study against SARS-CoV-2 D614G and SARS-CoV pseudoviruses. We found only one of the 23 expressed antibodies (mAb-12) effectively neutralized the D614G SARS-CoV-2 pseudovirus. mAb-12 showed a neutralization IC50 value of 0.087 μg/mL, and none of the antibodies showed neutralization against SARS-CoV (**Figure S5**). These data agree with other studies that show that antibody responses against SARS-CoV-2, and especially for cross-reactive antibodies, are predominantly non-neutralizing (76–78).

## Discussion

This study reveals a panel of naturally elicited antibody repertoire responses against SARS-CoV-2 from early in the pandemic, and mapped these antibodies against future emergent strains to understand B cell immune memory and cross-reactivity features. New SARS-CoV-2 variants have continued to evolve throughout the pandemic, and a novel coronavirus with high transmissibility and high fatality rate could possibly emerge (14, 15). The rapid rate of change of SARS-CoV-2 motivated our search to understand cross-reactive antibody responses and established immune memory against betacoronaviruses and SARS-CoV-2 variants. We combined a high-throughput single B-cell sequencing technology with yeast surface display and a massive antibody functional interpretation through NGS and bioinformatics to estimate affinity and functional antibody characteristics against diverse coronavirus probes.

In this study, we demonstrated that natively paired antibody yeast display screening can reliably detect antibody subdomain binding specificity and cross-reactivity at a large scale through NGS. Using this approach, we showed that 100% of the antibodies that were isolated and expressed were specific to SARS-CoV-2. Our data are consistent with previous studies that revealed a potential for immune evasion when patient libraries are exposed to SARS-CoV-2 variants (74, 79, 80). As in prior reports, we also confirmed that prior exposure to SARS-CoV-2 can generate substantial cross-reactive antibody responses against new SARS-CoV-2 variants and against broader coronaviruses (16, 23, 74, 76, 77). Our study leveraged next-generation sequencing and bioinformatic analysis to report a large panel of SARS-CoV-2–specific antibodies. Through this analysis, we report a total of 65 new cross-reactive monoclonal antibodies, and 28/65 (43%) of these showed substantial binding breadth against all SARS-CoV-2 variants evaluated in this study, and around 1/3 of the mAbs are cross-reactive against broader betacoronaviruses like WIV-1 and SCH014. We also used subdomain antigen screens and NGS-based bioinformatic mining to test epitope recognition of each variant. We found that about 40% of the antibodies in our panel showed binding to RBD and NTD subdomains, with the majority targeting conformational epitopes, or other non-RBD/non-NTD-specific epitopes on Spike protein. Prior reports showed that broadly cross-reactive anti-betacoronavirus antibodies can often bind to the S2 domain of Spike protein (45, 46, 76, 78, 81). Our data revealed that some non-RBD/non-NTD or conformation epitopes do recognize broadly, but also, we identified a number of RBD- and NTD-specific antibodies with broad recognition. We note that there was some difficulty in discerning clear signals in RBD/NTD sorts due to performing only one round of subdomain screening; future studies will use two rounds of subdomain screening to enhance data clarity. Additionally, we believe it is important to exclude the possibility of polyreactivity for antibodies that appear to target multiple antigens. To exclude mAb polyreactivity, we analyzed some broadly reactive antibodies by ELISA against SARS-CoV-2 antigens to verify appropriate recognition, and also against a model antigen to test for polyreactivity. None of the tested antibodies showed binding to non-specific model proteins, suggesting that the broadly reactive antibodies that we identified do bind specifically to broadly recognized Spike antigens.

Neutralization potency is also an important feature for protection against SARS-CoV-2 (54, 71, 81). We tested the potency of a subset of the antibodies discovered in this study, and we found that the vast majority of cross-reactive mAbs elicited here showed non-neutralizing activity. Our data are consistent with previous studies, which show that, even though virtually all patients develop neutralizing antibodies, around 35% of convalescent patients have low plasma levels of nAbs. These data suggest that other mechanisms of protection may play important roles in SARS-CoV-2 control, as for example Fc-mediated effector functions, including ADCC, ADCP, and opsonization (47, 48, 78, 82, 83). Healthy Fc effector function may be especially important for cross-reactive protection against future emergent strains *in vivo*, due to the limited cross-reactive neutralization elicited by infection with early SARS-CoV-2. To help understand developmental metrics for antibodies against SARS-CoV-2, we also performed a large repertoire-scale comparison of SARS-CoV-2 antigen-specific and non-specific antibodies according to the amount of SHM for each antibody. Our SHM analysis showed that even after convalescence from severe COVID-19, B cells targeting SARS-CoV-2 on average show less SHM than antibodies targeting non-SARS-CoV-2 antigens. These data suggest that the B cell response may be somewhat limited, impaired, or relatively less mature during early convalescence, which was the case for the donors in this study, compared to the average amount of SHM in patient-matched memory B cell populations.

Our study demonstrates an effective means for comprehensive mapping of the human immune repertoire against diverse SARS-CoV-2 and broadly recognized coronavirus probes, including the use of renewable screening libraries to evaluate immune memory of both current and future emergent pandemic variant strains that may not exist at the time of initial sample analysis. The results presented in this study can enhance the understanding of the functional profile of antibodies targeting SARS-CoV-2 antigens in COVID-19 convalescent patients, and provide new insights to help guide the discovery of new broadly reactive monoclonal antibodies and novel pan-betacoronavirus vaccines to protect against current and future SARS-CoV-2 variant strains.

## Data availability statement

The data presented in the study are deposited in the NCBI repository, accession number PRJNA862626

## Ethics statement

The studies involving human participants were reviewed and approved by Columbia University Irving Medical Center in New York, NY under IRB #AAAS9722. The patients/participants provided their written informed consent to participate in this study.

## Author contributions

MOS, BM, ITT, TZ, DDH, PDK, and BJD designed the experiments. MOS, BM, ITT, AH, LL, AF, SLA, XP and MS performed the experiments. MOS, BM, ITT, LL, AF, MGG, and BJD analyzed the data. MOS, BM, and BJD wrote the manuscript with feedback from all authors. All authors contributed to the article and approved the submitted version.

## Funding

This work was supported by NIH grants DP5OD023118 and R21AI143407 and by the Jack Ma Foundation, COVID-19 Fast Grants, the American Lung Association, and the Intramural Research Program of the Vaccine Research Center, National Institute of Allergy and Infectious Diseases, National Institutes of Health. MOS and AH were supported by the Gretta Jean & Gerry D. Goetsch Scholarship at the University of Kansas.

## Acknowledgments

We thank Jennifer Hackett for help with Illumina sequencing. **Figure 1A** was created with BioRender.

## Conflict of interest

The authors declare that the research was conducted in the absence of any commercial or financial relationships that could be construed as a potential conflict of interest.

## Publisher’s note

All claims expressed in this article are solely those of the authors and do not necessarily represent those of their affiliated organizations, or those of the publisher, the editors and the reviewers. Any product that may be evaluated in this article, or claim that may be made by its manufacturer, is not guaranteed or endorsed by the publisher.

## Supplementary material

Supplementary Figure 1 Yeast FACS analysis to analyze human antibody repertoire binding against SARS-CoV-2 antigenic S2P probes. Related to . Binding of yeast expressing SARS-CoV-2 libraries (Donor 1 and Donor 2), targeting the Spike protein (S2P) of SARS-CoV-2 variants WA-1, Alpha, Beta, Gamma, Delta, Epsilon, mutation D614G, and Beta-coronavirus Spike antigens WV1, SCH014, OC43, MERS, SARS-1, and HKU-1.

Supplementary Figure 2 Yeast FACS analysis to analyze human antibody repertoire binding against SARS-CoV-2 RBD and NTD subdomains probes. Related to . Binding of yeast expressing SARS-CoV-2 libraries (Donor 1 and Donor 2) targeting the RBD and NTD subdomains of SARS-CoV-2 variants Alpha, Beta, Gamma, Delta, and Epsilon.

Supplementary Figure 3 Enrichment ratio and ELISA binding analyses. Comparison between ELISA EC_50_ value and ER for anti-SARS-CoV-2 mAbs against SARS-CoV-2 D614G S2P.

Supplementary Figure 4 Polyreactivity analysis for selected broadly reactive anti-SARS-CoV-2 monoclonal antibodies. Binding profile of SARS-CoV-2-targeting mAbs 9 and 22 against SARS-CoV-2 NTD and D614G RBD, and to lysozyme from chicken egg white that was used as a polyreactive ligand. The anti-HIV mAb VRC01 was used was a negative control, and anti-SARS-CoV-2 mAbs 910-30 and Lp5 were used as positive controls for RBD and NTD respectively. Data are represented as mean ± SEM.

Supplementary Figure 5 Neutralization profiles of expressed mAbs. Pseudovirus neutralization for selected monoclonal antibodies against SARS-CoV-2 D614G, and also against the SARS-CoV betacoronavirus.
