# Supplementary figures and images for: Mapping monoclonal anti-SARS-CoV-2 antibody repertoires against diverse coronavirus antigens
Source: Front Immunol. 2022 Sep 2;13:977064. doi: 10.3389/fimmu.2022.977064 (PMC9478573; doi:10.3389/fimmu.2022.977064)

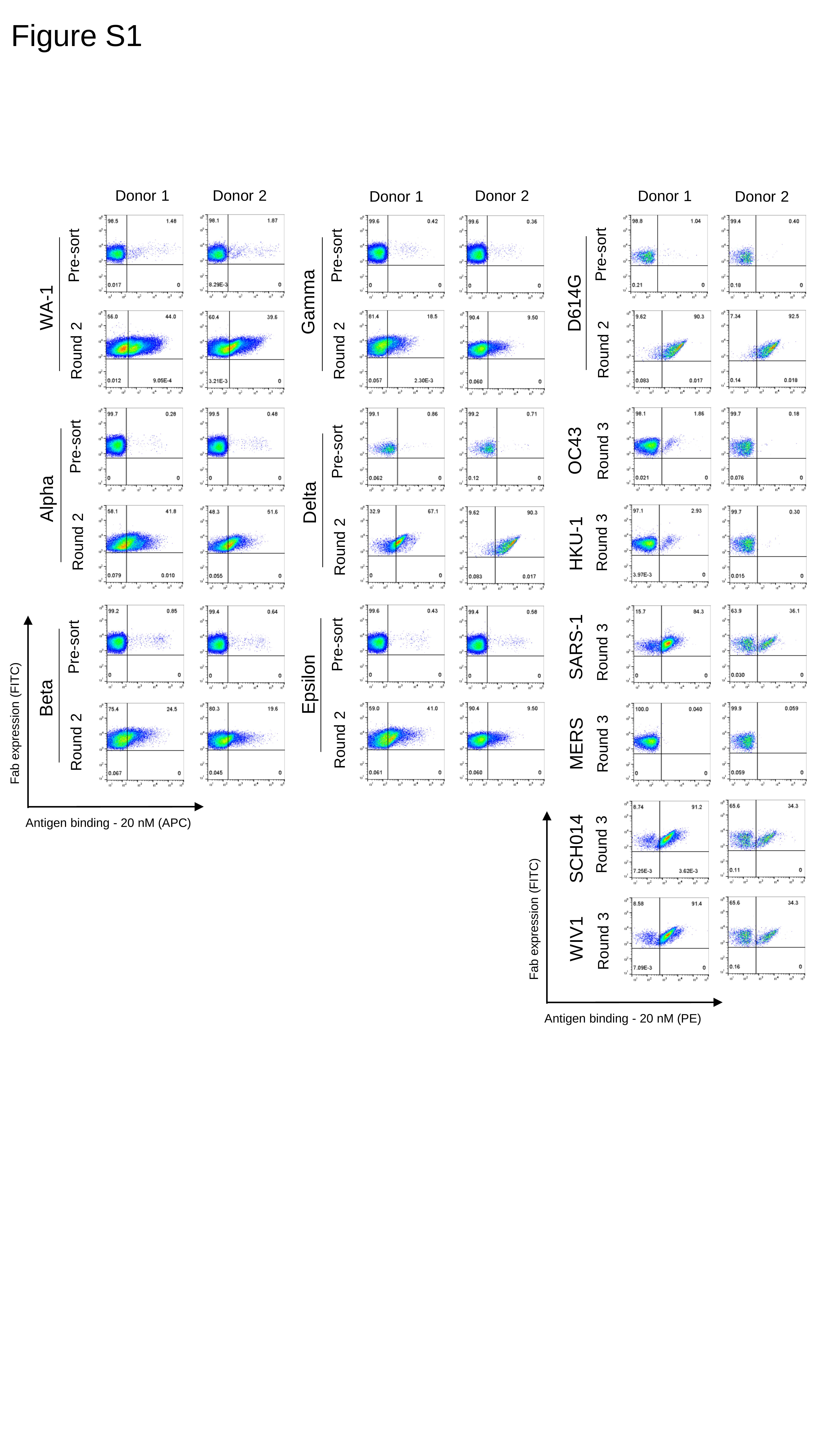

Supplement: Supplementary Figure 1 — Yeast FACS analysis to analyze human antibody repertoire binding against SARS-CoV-2 antigenic S2P probes. Related to . Binding of yeast expressing SARS-CoV-2 libraries (Donor 1 and Donor 2), targeting the Spike protein (S2P) of SARS-CoV-2 variants WA-1, Alpha, Beta, Gamma, Delta, Epsilon, mutation D614G, and Beta-coronavirus Spike antigens WV1, SCH014, OC43, MERS, SARS-1, and HKU-1. [file Image_1.jpeg]

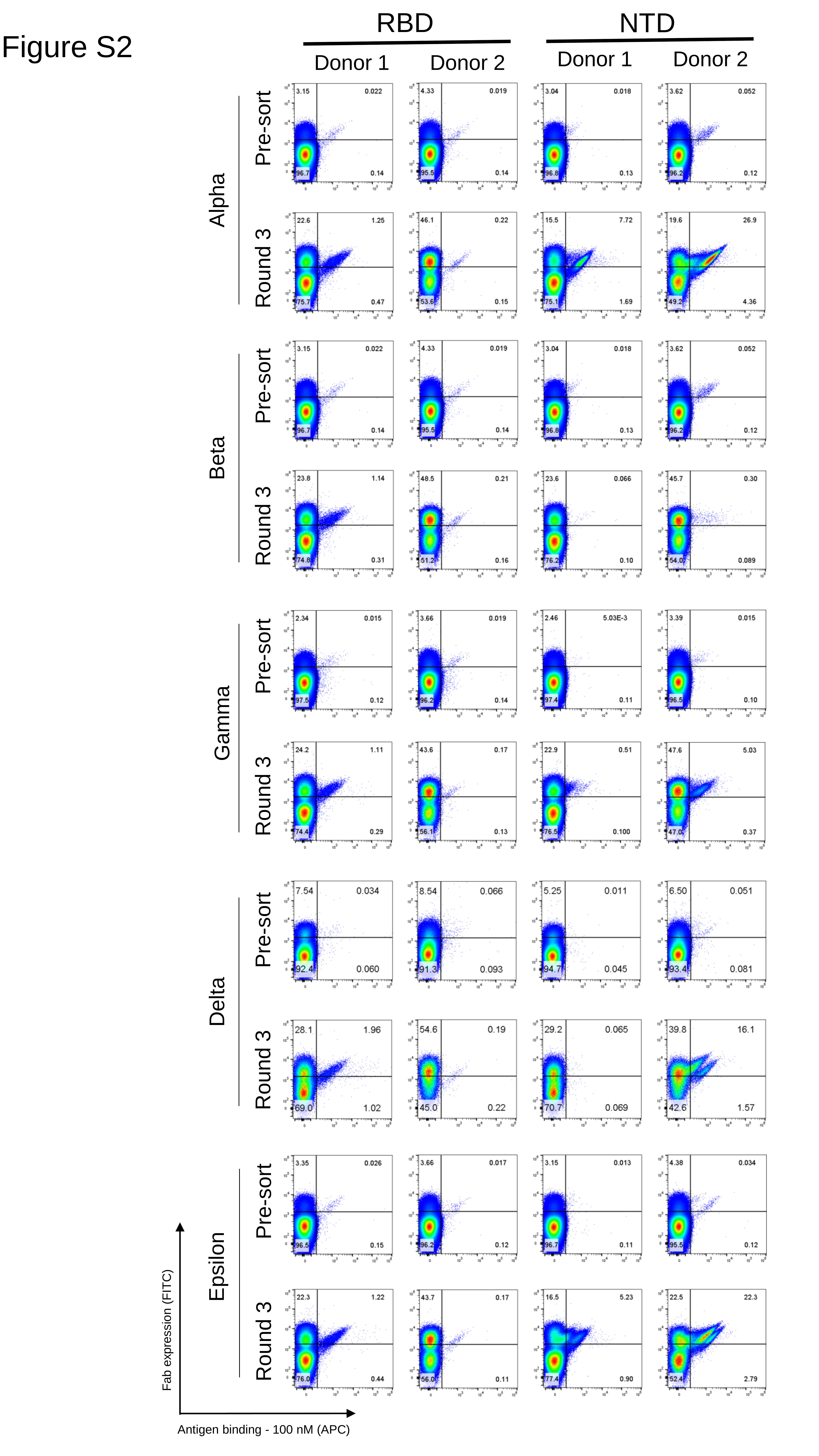

Supplement: Supplementary Figure 2 — Yeast FACS analysis to analyze human antibody repertoire binding against SARS-CoV-2 RBD and NTD subdomains probes. Related to . Binding of yeast expressing SARS-CoV-2 libraries (Donor 1 and Donor 2) targeting the RBD and NTD subdomains of SARS-CoV-2 variants Alpha, Beta, Gamma, Delta, and Epsilon. [file Image_2.jpeg]

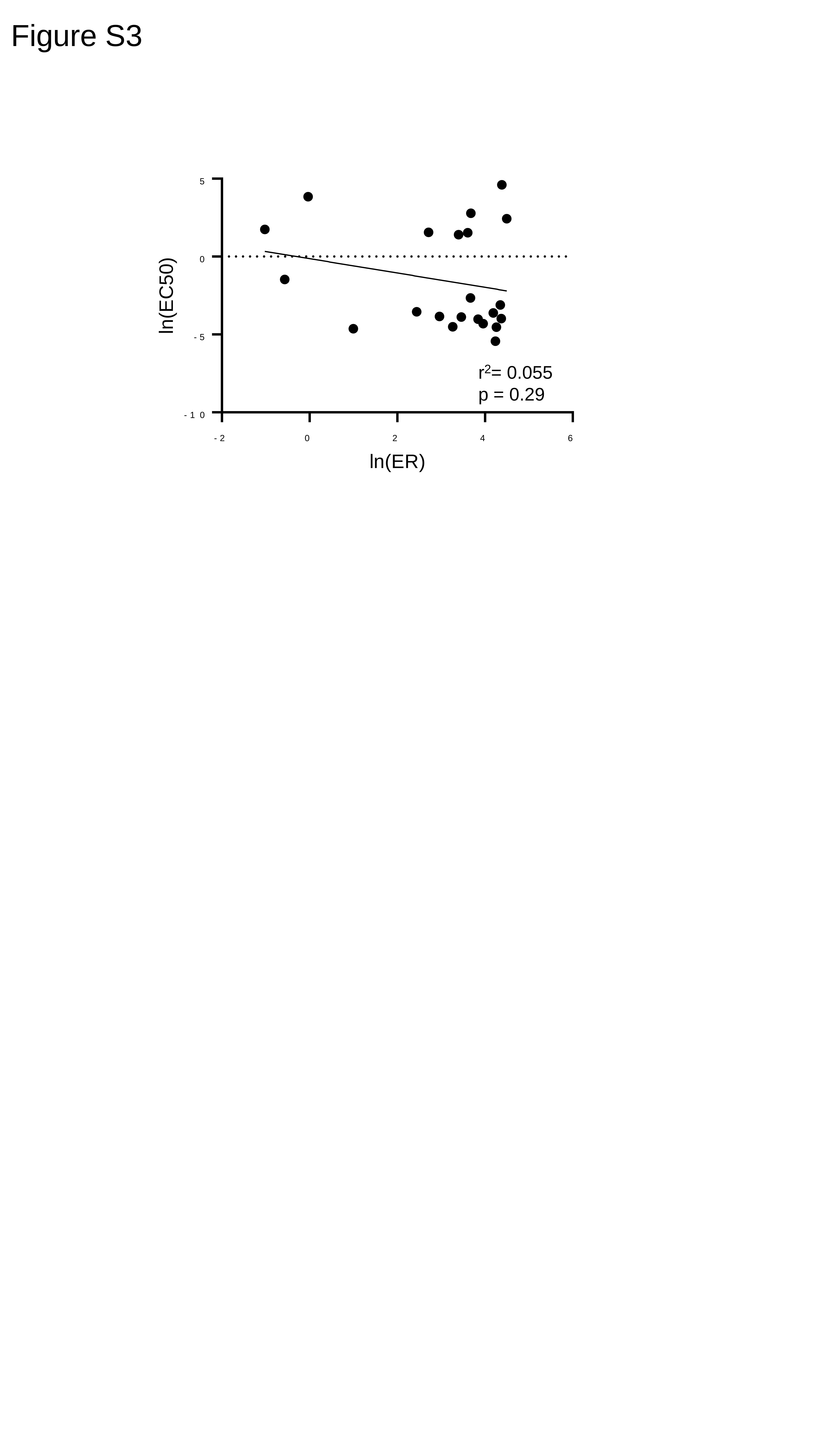

Supplement: Supplementary Figure 3 — Enrichment ratio and ELISA binding analyses. Comparison between ELISA EC50 value and ER for anti-SARS-CoV-2 mAbs against SARS-CoV-2 D614G S2P. [file Image_3.jpeg]

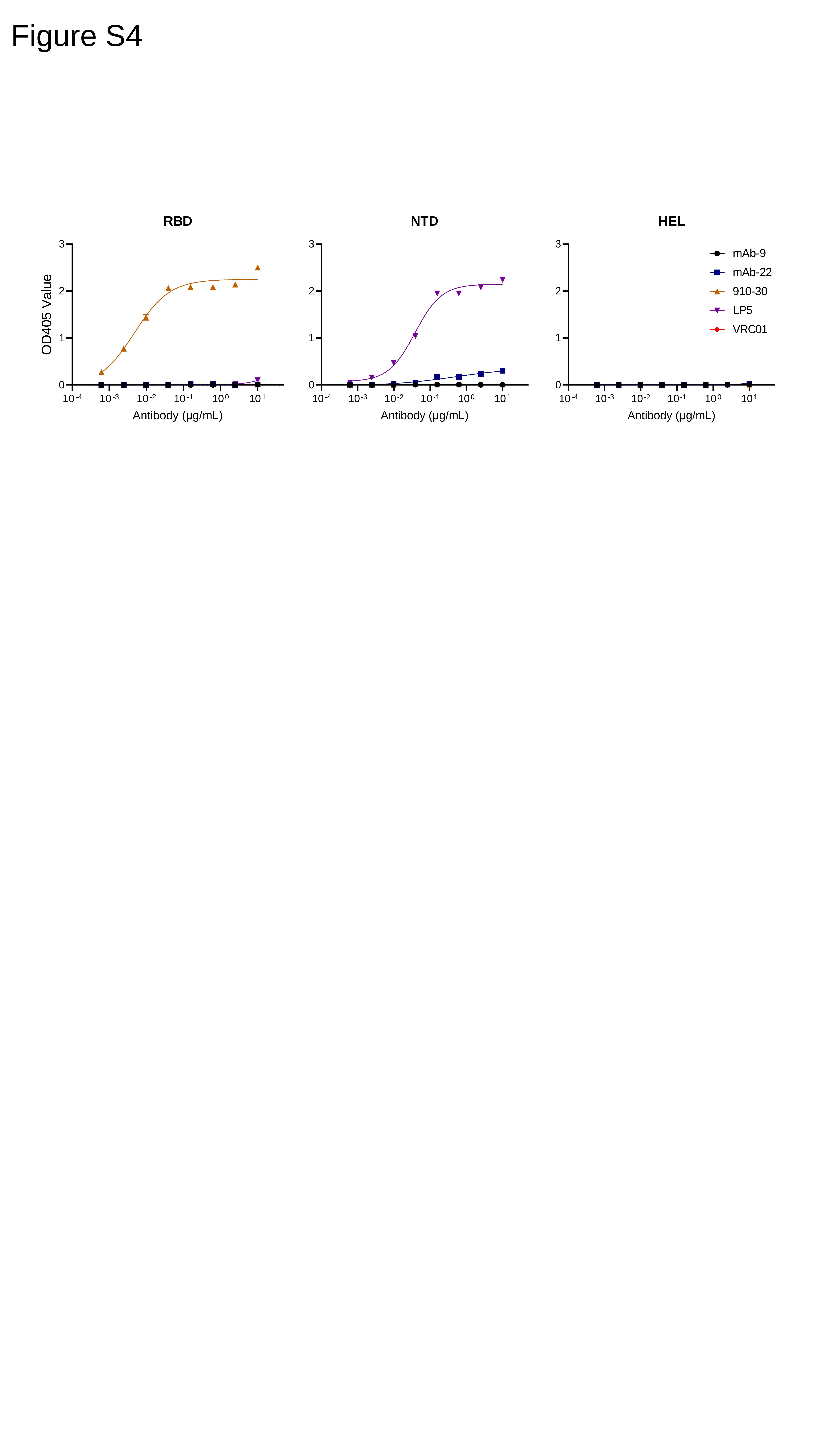

Supplement: Supplementary Figure 4 — Polyreactivity analysis for selected broadly reactive anti-SARS-CoV-2 monoclonal antibodies. Binding profile of SARS-CoV-2-targeting mAbs 9 and 22 against SARS-CoV-2 NTD and D614G RBD, and to lysozyme from chicken egg white that was used as a polyreactive ligand. The anti-HIV mAb VRC01 was used was a negative control, and anti-SARS-CoV-2 mAbs 910-30 and Lp5 were used as positive controls for RBD and NTD respectively. Data are represented as mean ± SEM. [file Image_4.jpeg]

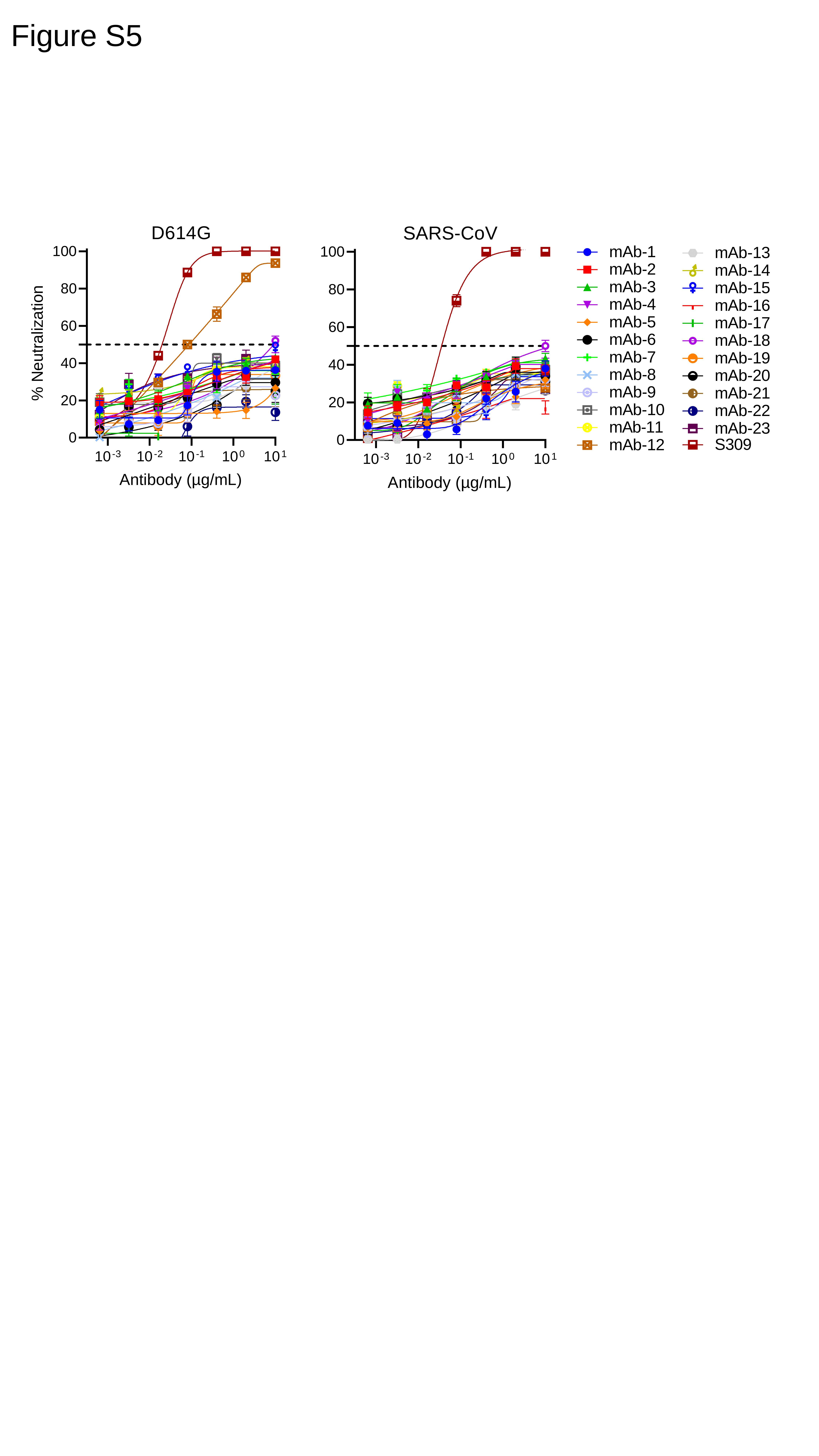

Supplement: Supplementary Figure 5 — Neutralization profiles of expressed mAbs. Pseudovirus neutralization for selected monoclonal antibodies against SARS-CoV-2 D614G, and also against the SARS-CoV betacoronavirus. [file Image_5.jpeg]
